# Decoding the mystery: AI-assisted bioinformatics and functional genomics technologies in medicinal plants

**DOI:** 10.3389/fpls.2025.1678483

**Published:** 2025-10-01

**Authors:** Cheng Song, Irfan Ali Sabir, Wanli Zhao, Yunpeng Cao

**Affiliations:** ^1^ College of Biological and Pharmaceutical Engineering, West Anhui University, Luan, China; ^2^ Key Laboratory of Biology and Genetic Improvement of Horticultural Crops, Ministry of Agriculture and Rural Affairs, College of Horticulture, South China Agricultural University, Guangzhou, China; ^3^ Institute of Botany, Jiangsu Province and Chinese Academy of Sciences, Nanjing, China; ^4^ State Key Laboratory of Plant Diversity and Specialty Crops, Wuhan Botanical Garden, Chinese Academy of Sciences, Wuhan, China

**Keywords:** bioinformatics, functional genomics, artificial intelligence - AI, machine learning, large language model

## Introduction

For millennia, medicinal plants have been a cornerstone of human healthcare, providing a rich source of bioactive compounds used in both traditional and modern medicine. A diverse array of therapeutic molecules is offered by these plants, from the antimalarial artemisinin in *Artemisia annua* to the anticancer alkaloids in *Catharanthus roseus*. The integration of artificial intelligence (AI) with bioinformatics and functional genomics has revolutionized the study of these medicinal plants, enabling researchers to explore their genetic and molecular underpinnings with unprecedented accuracy. These integrated technologies are transforming the study of medicinal plants, including drug discovery, responses to abiotic stresses, and the therapeutic potential of sustainable healthcare. However, the complexity and volume of genomic data pose significant challenges, necessitating advanced computational tools. AI, incorporating machine learning (ML) and deep learning (DL) techniques, has emerged as a powerful solution, capable of processing large volumes of data, identifying patterns and making predictions that traditional methods cannot match. This opinion explores several areas in which AI models in bioinformatics and functional genomics analysis are transforming medicinal plant research. Through detailed discussions and an exploration of future trends, we highlight how AI is reshaping our approach to medicinal plants, offering new possibilities for drug development and sustainable agriculture.

ML is considered a core technology in AI. Standard ML methods are overly narrow in their application to complex, natural, and high-dimensional raw data like genomic data. In contrast, DL methods are a promising and exciting area currently being widely applied in genomics, with successful applications in image recognition, audio classification, natural language processing, online web tools, chatbots, and robotics (Alharbi and Rashid, 2022). In this regard, DL as a genomics method is well-suited for analyzing large amounts of data. Although DL is still in its infancy in genomics, it holds the potential to transform fields such as clinical genetics and functional genomics. Multiple genomic fields are leveraging the generation of high-throughput data and harnessing the power of deep learning algorithms to make complex predictions. Modern advances in DNA/RNA sequencing technologies and machine learning algorithms, particularly deep learning, have opened up a new chapter in research, enabling the translation of large biological datasets into new knowledge and discoveries across various subfields of genomics (Lee, 2023). In the field of next-generation sequencing, modern deep learning tools have been proposed to overcome the limitations of traditional interpretation pipelines (Alharbi and Rashid, 2022). It has demonstrated that combining the deep learning-based variant caller DeepVariant with traditional variant callers (such as SAMtools and GATK) can improve the accuracy scores of single-nucleotide variant and indel detection (Kumaran et al., 2019). DeepVariant relies on graphical differences in input images to perform the classification task of genetic variant calling from NGS short reads (Hall et al., 2024). It treats mapped sequencing datasets as images and transforms variant calling into an image classification task.

Functional genomics aims to reveal the roles of genes and their interactions in biological systems (Zhang Y, et al., 2025). Traditional methods, such as gene set enrichment analysis, rely on existing genomic databases and are relatively cumbersome and time-consuming. However, many intriguing biological questions often exceed the limitations of these databases, and the introduction of AI offers new possibilities for filling these gaps. AI is reshaping the traditional way genomics research is conducted. By utilizing large language models (LLMs), scientists can significantly reduce manual analysis time and rapidly identify gene functions and interactions (Lotter et al., 2024). AI systems can quickly examine vast volumes of genomic data in drug discovery to find biomarkers and gene mutations linked to disease. This accelerates the development of new drugs and increases the success rate of drug discovery. For example, AI can screen thousands of compounds within hours to identify the most likely effective drug candidates. PDGrapher can identify the multiple factors that contribute to disease in cells and predict treatment options that can restore healthy cell function. Focusing on multiple pathogenic drivers, PDGrapher can identify the genes most likely to transform diseased cells into a healthy state and recommend the best single or combination therapeutic targets. Results indicated that the tool not only accurately predicted known effective drug targets but also discovered several new potential candidates (Gonzalez et al., 2025). Compared to similar models, PDGrapher achieved 35% higher predictive accuracy and operated up to 25 times faster.

Genome annotation involves identifying genes and their functions within a genome. It is a critical step in understanding the genetic basis of the therapeutic properties of medicinal plants. Traditional annotation methods, which rely on sequence similarity to known genes, can be labor-intensive and ineffective when dealing with novel or divergent paralogs, which are prevalent in plant genomes. However, AI has introduced innovative solutions that use machine learning algorithms, such as support vector machines (SVMs) and Bayesian methods, to predict gene functions based on sequence features and expression patterns. For instance, SVMs have been employed to identify drought-resistance genes in *Arabidopsis thaliana*, establishing a model for analogous applications in medicinal plants (Murmu et al., 2024). A significant advancement in this field is the application of deep learning to predict protein structures. Developed by DeepMind, AlphaFold 2 has achieved remarkable accuracy in predicting protein structures from amino acid sequences, thereby transforming functional genomics (Jumper et al., 2021; McCall and Almudevar, 2012). In *Salvia miltiorrhiza*, the structures of key enzymes involved in tanshinone biosynthesis were predicted, which helped the rational design of enzymes to enhance the production of these cardiovascular disease-protecting compounds (Chang et al., 2019; Zhou et al., 2017). Similarly, the homology-based gene prediction has been used to identify genes involved in withanolide biosynthesis, which are key adaptogenic compounds (Agarwal et al., 2017; Hakim et al., 2025). AI is also advancing single-cell genomics, enabling the study of gene expression at the cellular level. Tools like SIMLR (Single-cell Interpretation via Multi-kernel Learning) address challenges such as low-coverage single-cell RNA sequencing data, facilitating the clustering and annotation of rare cell types (Wang et al., 2018). In *C. roseus*, some bioinformatic tools were applied to annotated genes involved in terpenoid indole alkaloid (TIA) biosynthesis, thereby enhancing our understanding of tissue-specific expression (Rai et al., 2022). Despite these advancements, challenges persist. Many medicinal plants have large, complex genomes, and comprehensive genomic data for rare species is often lacking. The interpretability of DL models also poses a hurdle, as understanding their predictions is crucial for gaining biological insights. Ongoing efforts to develop standardized datasets and understandable DL models are addressing these issues, and these efforts are promising to expand the application in genome annotation for medicinal plants.

Metabolic pathways are central to the production of secondary metabolites in medicinal plants. These are often responsible for their therapeutic properties. Reconstructing these pathways is essential for understanding biosynthesis and for engineering plants to produce more compounds (Song et al., 2022a). Bioinformatics and genomics have transformed this process by combining metabolomics data with sophisticated computational methods. Machine learning algorithms predict metabolic pathways by analyzing metabolite concentrations and gene expression patterns. For instance, metabolic engineering helps to reconstruct the artemisinin biosynthetic pathway in *A. annua*, identifying key genes and enzymes, thereby informing strategies to increase artemisinin yields (Costello and Martin, 2018). Gene mining is the process of identifying genes of interest from genomic data. This is another area where AI excels. ML models classify genes based on sequence and expression data, pinpointing those involved in metabolite production. In *Panax ginseng*, the glycosyltransferases (*UGTs*) and *CYP450* family genes responsible for ginsenoside production, paving the way for genetic engineering to boost ginsenoside content (Hou et al., 2021; Xu et al., 2017). Similarly, large-scale gene mining in *C. roseus* genome has shed light on the biosynthesis of TIAs, which are vital anti-cancer agents (McCall and Almudevar, 2012). AI facilitates the discovery of novel pathways. By analyzing multi-omics datasets, we can predict pathways that are not apparent through traditional methods, particularly in understudied plants. In *Ophiorrhiza pumila*, some key genes involved in camptothecin biosynthesis were identified by integrating transcriptomic and metabolomic data (Yang et al., 2021). Tools such as ClusterFinder and DeepBGC use hidden Markov models (HMMs) and DL method to identify biosynthetic gene clusters (BGCs), which are essential for producing secondary metabolites (Liu et al., 2022; Hannigan et al., 2019). Genome-wide identification of *WRKY* members from *Myrica rubra* revealed that the *WRKY14* significantly activates the promoter region of the *SWEET1* gene, suggesting its positive regulatory role in sugar synthesis (Fan et al., 2025). These advancements would have a lasting effect on drug discovery and agricultural biotechnology by enabling targeted genetic modifications to optimize the production of therapeutic compounds. However, challenges such as data scarcity for rare plants.

Integrated multi-omics data — including genomics, transcriptomics, proteomics, and metabolomics — provides a comprehensive view of plant biology (Song et al., 2022b; Zhang et al., 2023). Large language model facilitates this process by managing the complexity and volume of the data. The orthogonal projections to latent structures (OPLS) method can integrate transcriptomic and metabolomic data, and tools such as iDREM can construct integrated networks from temporal data (Kumar et al., 2024). In *S. lycopersicum*, multi-omics integration has optimized metabolic networks to improve fruit quality (Cembrowska-Lech et al., 2023). The optimization of metabolic networks involves predicting and manipulating pathways to increase the yield of therapeutic compounds. Challenges such as data noise, sparsity and scaling issues are being overcome. This is because of its ability to handle high-dimensional data. Predicting gene regulatory networks (GRNs) is essential for understanding how genes are regulated in response to environmental and developmental cues. AI, particularly neural network-based methods, predicts transcription factor binding sites and regulatory relationships. In *C. roseus*, AI has been used to predict networks involved in TIA biosynthesis and identify key regulators (Pan et al., 2016). Transformer-based models are used by tools like Enformer and RNABERT to predict genome interactions and RNA clustering, respectively (Avsec et al., 2021). These advancements facilitate the identification of new therapeutic targets and pathways, enhancing the potential for genetic engineering in medicinal plants. However, genetic modification raises ethical concerns, requiring careful assessment of ecological impacts.

GRNs govern gene expression in response to environmental and developmental signals. Advances in AI have led to the development of tools such as iDREM and GRNBoost2, which can construct temporal and cell-specific GRNs from multi-omics data (Sharma et al., 2024). These tools have been used to study stress responses in Arabidopsis, revealing complex regulatory mechanisms. In medicinal plants, predicting GRNs is crucial for understanding how therapeutic compounds are produced. For example, AI has been employed to predict the regulatory networks involved in TIA biosynthesis in *C. roseus*, identifying the transcription factors that control alkaloid production. Transfer learning has also enabled cross-species predictions, such as the identification of metabolism-related genes in *S. lycopersicum* (Badia-i-Mompel et al., 2023). In *Withania somnifera*, genome-wide identification has identified stress-responsive genes involved in withanolide biosynthesis, thereby enhancing plant resilience and compound yield (Nicolis et al., 2024; Tripathi et al., 2020). Gene co-expression network analysis is particularly valuable for identifying stress-related genes, as many secondary metabolites are produced in response to environmental stresses. By analyzing gene expression under various conditions, large language models can classify genes based on their stress responsiveness. This provides targets for breeding stress-tolerant medicinal plants. The complexity of GRNs and the need for comprehensive multi-omics data are just two of the challenges that must be overcome (Badia-i-Mompel et al., 2023; Otal et al., 2025). Using more sophisticated bioinformatics and data integration techniques is helping to resolve these issues and make predictions more accurate (Song et al., 2023; Zhang J, et al., 2025).

## Discussion

Despite the immense success of these tools in genomics and bioinformatics, the adoption of different DL solutions and models remains limited. One reason is the lack of published DL-based protocols that can adapt to new, heterogeneous datasets that require extensive data engineering. In genomics, high-throughput data are used to train neural networks and have become a typical approach for disease prediction or understanding regulatory genomics (Schmidt and Hildebrandt, 2021). Similarly, developing new DL models and testing existing models on new datasets are significant challenges due to the lack of comprehensive, generalizable, and practical biology-oriented deep learning libraries (Munappy et al., 2022). In this regard, software frameworks and genomic packages are crucial for quickly adopting new research questions or hypotheses, integrating raw data, or conducting research using different neural network architectures. Recently, advances in NLP and LLMs have improved data integration and analysis. GRNs can address data scarcity by generating synthetic datasets, and attention mechanisms can enhance model interpretability. Future breakthroughs will depend on interdisciplinary collaboration between biologists, computer scientists, and data scientists. Despite significant advancements, challenges remain in applying AI to medicinal plant research. Standardized datasets that include genomic, transcriptomic, proteomic, metabolomic and phenotypic data are essential for training robust AI models. A range of resources on spice genomics have been developed to help identify the most promising future directions. These resources include genome assemblies, sequencing and re-sequencing projects, as well as studies based on the transcriptome, non-coding RNA-mediated regulation, organelles-based resources, developed molecular markers, web resources, databases and AI-directed resources (Das et al., 2023). All of these are focused on enhancing the breeding potential of specific spices. While there are extensive datasets for model plants, many medicinal plants still lack sufficient genomic resources, which limits AI applications. Although deep learning models are highly accurate, they often operate like black boxes, hindering the translation of predictions into biological insights. Therefore, developing explainable AI models is crucial for gaining trust and extracting actionable biological insights. Additionally, the substantial computational resources required for genome-wide identification analyses present a challenge for researchers in settings with limited resources. One solution is to develop lightweight AI models for use in such environments, as well as using GRNs to create gene expression data for model training and integrating attention mechanisms to focus on biologically relevant features.
